# Dengue virus-like particles mimic the antigenic properties of the infectious dengue virus envelope

**DOI:** 10.1186/s12985-018-0970-2

**Published:** 2018-04-02

**Authors:** Stefan W. Metz, Ashlie Thomas, Laura White, Mark Stoops, Markus Corten, Holger Hannemann, Aravinda M. de Silva

**Affiliations:** 1 Department of Microbiology and Immunology, University of North Carolina, Chapel Hill, USA; 2 The Native Antigen Company, Kidlington, Oxford, UK

**Keywords:** Dengue virus, VLP, Epitopes, Serum depletions

## Abstract

**Background:**

The 4 dengue serotypes (DENV) are mosquito-borne pathogens that are associated with severe hemorrhagic disease. DENV particles have a lipid bilayer envelope that anchors two membrane glycoproteins prM and E. Two E-protein monomers form head-to-tail homodimers and three E-dimers align to form “rafts” that cover the viral surface. Some human antibodies that strongly neutralize DENV bind to quaternary structure epitopes displayed on E protein dimers or higher order structures forming the infectious virus. Expression of prM and E in cell culture leads to the formation of DENV virus-like particles (VLPs) which are smaller than wildtype virus particles and replication defective due to the absence of a viral genome. There is no data available that describes the antigenic landscape on the surface of flavivirus VLPs in comparison to the better studied infectious virion.

**Methods:**

A large panel of well characterized antibodies that recognize epitope of ranging complexity were used in biochemical analytics to obtain a comparative antigenic surface view of VLPs in respect to virus particles. DENV patient serum depletions were performed the show the potential of VLPs in serological diagnostics.

**Results:**

VLPs were confirmed to be heterogeneous in size morphology and maturation state. Yet, we show that many highly conformational and quaternary structure-dependent antibody epitopes found on virus particles are efficiently displayed on DENV1–4 VLP surfaces as well. Additionally, DENV VLPs can efficiently be used as antigens to deplete DENV patient sera from serotype specific antibody populations.

**Conclusions:**

This study aids in further understanding epitopic landscape of DENV VLPs and presents a comparative antigenic surface view of VLPs in respect to virus particles. We propose the use VLPs as a safe and practical alternative to infectious virus as a vaccine and diagnostic antigen.

**Electronic supplementary material:**

The online version of this article (10.1186/s12985-018-0970-2) contains supplementary material, which is available to authorized users.

## Background

Dengue viruses (DENV) are mosquito-borne flaviviruses responsible for dengue fever and dengue hemorrhagic fever [1, 2]. DENVs are endemic in over 125 countries and its global distribution is expected to expand due to urbanization and other environmental factors that favor the mosquito vector [3]. The DENV complex consists of 4 antigenically distinctive serotypes (DENV1–4). Infectious DENV particles contain a positive stranded RNA genome and capsid proteins surrounded by a lipid envelope, which has two membrane glycoproteins designated as pre-membrane (prM) and envelope (E). The outer surface of the mature, infectious virus has a smooth surface covered by E protein homodimers [4]. On the viral surface, units of three E protein homodimers assemble into raft-like structures and 30 rafts are packed tightly to create a protein coat with icosahedral symmetry. The E protein mediates viral attachment and entry into cells. Additionally, the E protein is the major target of neutralizing and protective human antibodies. Recent studies have demonstrated that most human antibodies that strongly neutralize DENVs bind to quaternary structure E protein epitopes containing regions from 2 or more proteins packed on the viral surface [5–8]. In other words, many human antibodies target E protein epitopes displayed on the viral surface but not on individual E protein subunits. Soluble E protein subunit antigens are, therefore, considered to be poor vaccine immunogens because they lack higher order protein structures and epitopes displayed on the intact virus. When flavivirus prM and E proteins are co-expressed in cells, these proteins assemble into virus-like particles (VLPs) that are secreted from cells. Flavivirus VLPs have been produced in a wide variety of expression platforms such as plants, insect cells, bacteria, yeast and mammalian cells. Flavivirus VLPs, which do not contain a viral nucleocapsid or genomic RNA, are smaller than intact virions [9–12]. VLPs, often, share structural similarities and physicochemical features of the native virus, but are superior in terms of safety, and ease of production and purification [10]. VLPs have proven to be effective vaccine antigens in preclinical or early stage clinical studies with different enveloped viral pathogens such as West Nile virus, Tick-borne encephalitis virus, Japanese encephalitis virus, Zika virus, Chikungunya virus, Influenza virus and Dengue virus [10–17]. For DENV VLPs, no data is available on the display and presentation of epitopes of varying complexity recognized by human neutralizing antibodies. Some structural information is available for tick-borne encephalitis virus (TBEV) VLPs [12]. The low-resolution cryo-EM image based reconstruction of TBEV VLPs indicates that the number and organization of E proteins on the VLPs are different from the infectious virus. Even though TBEV VLPs are heterogeneous in size, investigators analyzed a uniform population of particles that were smaller than virions and concluded that VLPs contain 30 E-homodimers assembled in a *T* = 1 icosahedral lattice [12]. It is unclear if quaternary structure antibody epitopes, especially those containing residues from different adjacent E homo-dimers, are displayed similarly on VLPs and the larger infectious virions [12].

In this study we describe in detail the properties of epitopes present on DENV VLPs of all 4 serotypes using a large panel of well-defined human mAbs and immune sera from dengue patients. We directly compare the antigenic features of DENV VLPs to whole virions and explore their use as tools in serologic assays. Our results show the equivalence between viruses and VLPs in a setting that is particularly relevant for flavivirus vaccine development, diagnostics and understanding the difference between virus and VLPs.

## Methods

### Production of DENV VLPs

#### Cells and viruses

Vero-81 cells were maintained at 37 °C as a monolayer in Dulbecco’s modified Eagle’s medium (DMEM, Gibco) supplemented with 5% fetal bovine serum (FBS), 1% non-essential amino acids, 100 U/ml penicillin and 100 μg/ml streptomycin. Culture supernatants of DENV1 WestPac-74, DENV2 S-16803, DENV3 CH53489 and DENV4 TVP-376 virus strains were used to determine antibody binding and neutralizing activity of depleted and undepleted serum. Purified DENV virus preparations were obtained according to previously described protocols [18] .

#### DENV virus-like particle production

The DENV virus-like particles (VLPs) were produced and kindly provided by The Native Antigen Company, Kidlington, UK. In short: recombinant dengue virus-like particles consisting of DENV prM and E proteins were transiently expressed in suspension culture adapted HEK293 cells. Three days post transfection culture supernatant was cleared by centrifugation and concentrated by tangential flow filtration. In a first step VLPs were purified by a 2-step discontinuous sucrose gradient with 20% and 40% sucrose densities. After a 6 h spin at 25,000 rpm in a SW28 rotor at 10 °C the 20%–40% interphase was harvested by needle-stick. As a second purification step ion exchange chromatography (Q Sepharose High Performance; GE Healthcare) was used in negative mode once. VLPs were further purified by size exclusion chromatography (Toyopearl HW-65F) which also provided exchange of buffers to storage buffer. Purified DENV1–3 VLPs were stored in 10 mM sodium phosphate, 20 mM sodium citrate, 154 mM sodium chloride, pH 7.4. DENV4 VLPs were stored in Dulbecco’s phosphate buffered saline (DPBS) pH 7.4 containing 30% sucrose. VLP samples were stored at − 80 °C.

#### Virus and VLP analysis by western blot

Purified VLP samples were subjected to sodium dodecyl sulphate polyacrylamide electrophoresis (SDS-PAGE) and analyzed by western blot (WB) and Coomassie Brilliant Blue (CBB) staining. 500 ng of DENV VLP or purified DENV virus [18] were loaded into a gel loading buffer (Biorad) supplemented with SDS. Samples were boiled for 10 mins at 95 °C and subsequently spun down at full speed for 2 mins. Following electrophoresis, separated protein fractions were transferred to a nitrocellulose membrane and blocked in blocking buffer (3% skim milk in phosphate buffered saline (PBS) + 0.05% Tween-20). Blocked membranes were incubated with 0.5 μg/ml 1 M7 mAb or 2G3 mAb in blocking buffer for 1 h at 37 °C. Next, membranes were washed and treated with (1:1000 in blocking buffer) AP-conjugated anti-human IgG for 1 h at 37 °C. After washing, membranes were developed by NBT/BCIP AP substrate (Thermo Scientific).

#### Epitope analysis by enzyme-linked immunosorbent assay

100 ng/well of 1M7 or 4G2 mAb was coated onto High-binding Microlon plates (Greiner) in 0.1 M bicarbonate buffer pH 9.6 overnight at 4 °C. Plates were washed 3 times with PBS + 0.1% Tween-20 and blocked with PBS + 3% skim milk + 0.05% Tween-20 for 1 h at 37 °C. Next, plates were washed and incubated with serially diluted purified virus or VLPs (of 2 separate preparations) in blocking buffer for 1 h at 37 °C. After washing, the 1 M7 coated plates were incubated with the indicated serially diluted mouse derived mAbs and the 4G2 coated plates were incubated with indicated serially diluted human mAbs (Table 1) for 1 h at 37 °C. After washing, mouse mAb treated wells were incubated with 1:1000 anti-mouse IgG-AP conjugated (Sigma) and human mAb treated wells were incubated with 1:2500 anti-human IgG-AP conjugated (Sigma). Plates were subsequently washed and developed using AP-substrate (Sigma). Absorbance was measured at 405 nm.

**Table 1:** Monoclonal antibody characterization

mAb/ polyclonal sera	M/H/C	Binding	Neutralization *(W/M/S)*	Binding region	Binding DENV serotypes					Ref
					*DV1*	*DV2*	*DV3*	*DV4*	ZIKV	
*4G2*	M	F-CR	W	DII FL	++	++	+++	+++	+++	[26]
*1F4*	H	DV1	DV1:S	DI/DII hinge Q	+++	–	–	–	–	[7]
*12C1.5*	M	D-CR	DV:S	DIII	++	++	+++	++	–	[27]
*1C19*	H	D-CR	DV:M	DII FL/BC	++	++	++	+	++	[28]
*2D22*	H	DV2	DV2:S ZIKV:W	DII/DIII Q	–	++	–	–	–	[5, 29]
*5J7*	H	DV3	DV3:S	DI/DII Q	–	–	+++	–	–	[23]
*5H2*	C	DV4	DV4:S	DI Q	–	–	–	+	–	[24]
*1 M7*	H	F-CR	M	DII FL	+++	++	+++	+++	+++	[30]
*A11 (EDE2)*	H	F-CR	DV:S ZIKV:W	DI/DII/DIII Q	+++	+++	+++	+++	+	[31]
*B7 (EDE2)*	H	F-CR	DV:S ZIKV:W	DI/DII/DIII Q	+++	+++	+++	+++	+	[31]
*C8 (EDE1)*	H	F-CR	DV:S ZIKV:S	DII/DIII Q	+++	+++	+++	+++	++	[31]
*C10 (EDE1)*	H	F-CR	DV:S ZIKV:S	DII/DIII Q	+++	+++	+++	+++	++	[31]
*3H5*	M	DV2	DV2:S	DIII LR	–	+++	–	–	–	[32]
*8A1*	M	DV3	DV3:S	DIII	–	–	+++	–	–	[33]
*DV4 126*	H	DV4	DV4:S	DI/DII hinge Q	–	–	–	+++	–	[8]
*DV4 131*	H	DV4	DV4:S	DI/DII hinge Q	–	–	–	+++	–	[8]
*DV4 141*	H	DV4	None	DIII	–	–	–	+++	–	[8]
*DT000*	H	D-CR	DV:S ZIKV:W		+++	+++	+++	++	–	[21]

Dengue specific monoclonal antibodies. A panel of well-defined mouse, human or chimpanzee (M/H/C) derived Mabs were used to characterize sRecE epitopes. Flavivirus cross reactive (F-CR), dengue cross reactive (D-CR), weakly, moderately or strong (W/M/S) neutralizing, E-domain I, II, III (DI, DII, DIII), fusion loop (FL), BC-loop (BC), lateral ridge (LR), quaternary (Q)

The relative epitope display of each mAb tested was determined for both the purified virus as the VLPs of each serotype. The binding of all mAbs was normalized against the binding of 1F4 for DENV1, 2D22 for DENV2, 5 J7 for DENV3 and 5H2 for DENV4 to determine the relative epitope display of all other mAbs. The normalized increase or decrease in mAb binding was compared between virus and VLPs to give an indication of which epitopes are displayed better on the virus and VLP.

#### DT000 serum depletion

DT000 sera was derived from an individual > 10 years post a secondary DENV infection (Table 1) and was diluted 1:10 in PBS and subjected the serum depletion using DENV purified virus antigen or DENV VLPs (The Native Antigen Company). 100 μg of 1 M7 mAb was conjugated to 10 mg of Tosylactivated M280 magnetic Dynabeads (Invitrogen) using manufacturers protocol. 1 M7 coated beads were blocked with 1% bovine serum albumin (BSA) in PBS for 1 h at 37 °C. The beads were washed 3 times with blocking buffer and incubated on a rotator with 100 μg in 500 μl blocking buffer of purified DENV antigen, DENV VLPs or BSA for 1 h at 37 °C. Next, the beads were washed 3 times with PBS and cross-linked with 4% PFA in 250 μl PBS for 30 mins at room temperature. Next, the beads were washed 4 times in PBS, resuspended in 100 μl PBS and subsequently divided over two tubes for two rounds of depletion. 500 μl of diluted serum was incubated with the beads for 1 h at 37 °C on a rotator. Next, the serum was transferred to the second tube of beads for the second round of depletion. Finally, the depleted serum was stored at 4 °C for further analysis by ELISA and flow based vero cell neutralization assay.

#### DENV specific IgG ELISA

Microlon ELISA plates (Greiner) were coated with 100 ng/well of 4G2 mAb in 0.1 M bicarbonate buffer pH 9.6 overnight at 4 °C. The plates were blocked with PBS + 3% skim milk + 0.05% Tween-20 for 1 h at 37 °C. Next, the plates were washed 3 times with PBS + 1% Tween-20 and incubated with 1:10 diluted virus culture supernatant in blocking buffer for 1 h at 37 °C. After washing, the plates were incubated with depleted or undepleted DT000 or DT168 serum (final concentration 1:50 diluted in blocking buffer) for 1 h at 37 °C. Next, the plates were washed and incubated with 1:2500 anti-human IgG-AP conjugated (Sigma) for 45 mins at 37 °C. The plates were washed and developed with AP-substrate (Sigma) and the absorbance was measured at 405 nm.

#### Vero cell neutralization assay

The neutralizing capacity of depleted and undepleted DT000 serum was determined by flow based neutralization assay as previously described [19, 20]. In short: 25000 Vero cells/well were seeded in a 96-wells flat-bottom culture plate and incubated overnight at 37 °C. Depleted and undepleted sera were serially diluted in OptiMem (Gibco) and incubated for 45 mins at 37 °C with the appropriate amount of virus (resulting in 15% of infected cells - amount previously determined). Cells were washed with OptiMem and overlayed with the virus/serum mix for 2 h at 37 °C. Next, cells were washed and overlaid with Vero cell growth medium and incubated overnight at 37 °C. The next day, cells were detached using trypsin (Gibco) and transferred to a 96-wells round bottom well plate. Cells were fixed in 4% paraformaldehyde in PBS for 10 mins at room temperature. Cells were subsequently washed in permeabilization buffer and blocked with 1% normal mouse serum in PBS for 30 mins at room temperature. The cells were treated with Alexa fluor 488 conjugated anti-prM Mab 2H2 and diluted 1:400 in blocking buffer for 1 h at 37 °C. Cells were washed in permeabillization buffer and finally resuspended in PBS + 1% FBS. The percentage of infected cells was determined by flow cytometry using a Guava® Flow Cytometer (EMD Millipore) and the neutralizing efficiency of the sera was expressed as neut_50_ values (the dilution where 50% of the virus was neutralized) calculated using GraphPad Prism software.

### Transmission electron microscopy

Discharged copper 400 mesh formvar carbon coated grids (Ted Pella Inc., Redding, CA, USA) were loaded with 15 μl of purified DENV VLPs for 5 mins at room temperature. The grids were washed in MilliQ water and stained for 30 s with 2% uranyl acetate. Excess uranyl acetate was removed by filter paper. The grids were finally air-dried at RT and observed with a LEO 910 transmission electron microscope (Zeiss).

## Results

### Expression and purification of DENV VLPs

**Fig. 1:**
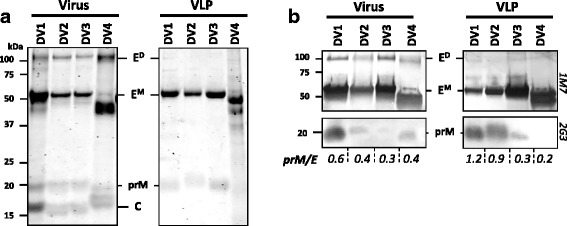
Expression and characterization of DENV1–4 VLPs. Purified virus and VLPs were subjected to SDS-PAGE and analyzed with (**a**) CBB and (**b**) WB using anti-E 1 M7 and anti prM 2G3 mAbs. E-dimers (E^D^), E-monomers (E^M^), prM and capsid (**c**) proteins are indicated. The prM/E ratio is indicated below panel B and is determined using ImageJ software

DENV1–4 prM-E sequences were expressed in mammalian HEK 293 suspension cultures and purified by sucrose gradient and ion-exchange chromatography. Purified VLP fractions were subjected to SDS-PAGE and analyzed by Coomassie stain (CBB; Fig.1a) and Western Blot (WB) using a cross-reactive (CR) human derived anti-E mAb 1 M7 and anti-prM 2G3 (Fig. 1b). All virus and VLP samples showed a protein band of ~ 55 kDa, corresponding to the estimated molecular weight of the DENV E protein. There were some slight differences observed in molecular weight between the 4 serotypes, possibly due to differences in the number of N-linked glycans. However, the size differences were consistent for the virus samples and the VLP samples. The levels of prM seemed to differ between purified virus and VLP samples. DENV1 and DENV2 VLPs appear to contain more prM than their virus counterpart, while DENV4 VLPs had lower prM levels compared to DENV4 purified virus as determined by the prM/E ratio. However, the difference in prM content and maturation differences between VLP serotypes need to be further analyzed in other more focused studies. E-dimer fractions appeared more prominent in the virus samples as compared to VLPs.

**Fig. 2:**
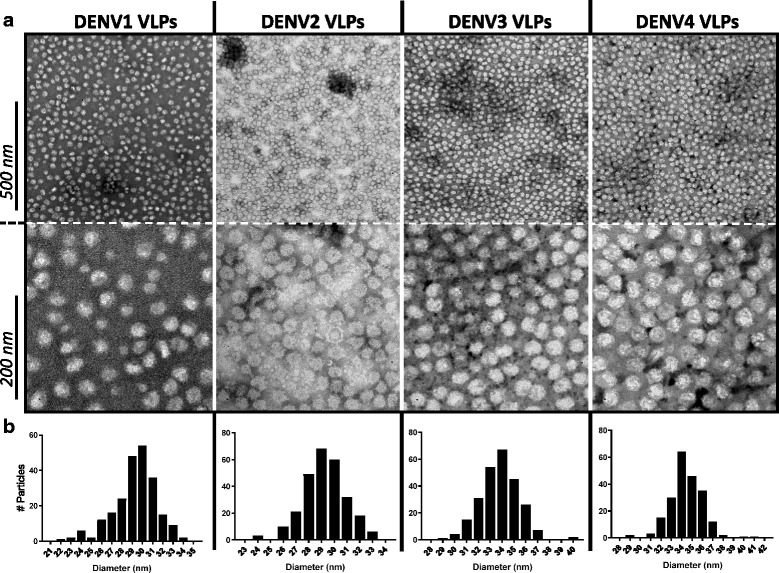
TEM analysis of DENV1–4 VLPs. **a** Particle shape and distribution was analyzed by TEM using negative staining. **b** VLP size distribution was determined by measuring the diameter of ~ 250 particles of each serotype

Negative staining followed by transmission electron microscopy (TEM) revealed that the DENV VLPs are fairly monodispersed, but heterogeneous in morphology and size (Fig. 2). The VLPs appear as semi-smooth spherical particles, with noticeable roughness at their surface. The diameter of ~ 250 VLPs of each serotype shows a size distribution between 25 and 40 nm, with DENV3 and DENV4 VLPs being slightly bigger (~ 34–35 nm) in size compared to DENV1 and DENV2 VLPs (~ 29–30 nm).

### DENV VLPs display similar epitopes than native virus

**Fig. 3:**
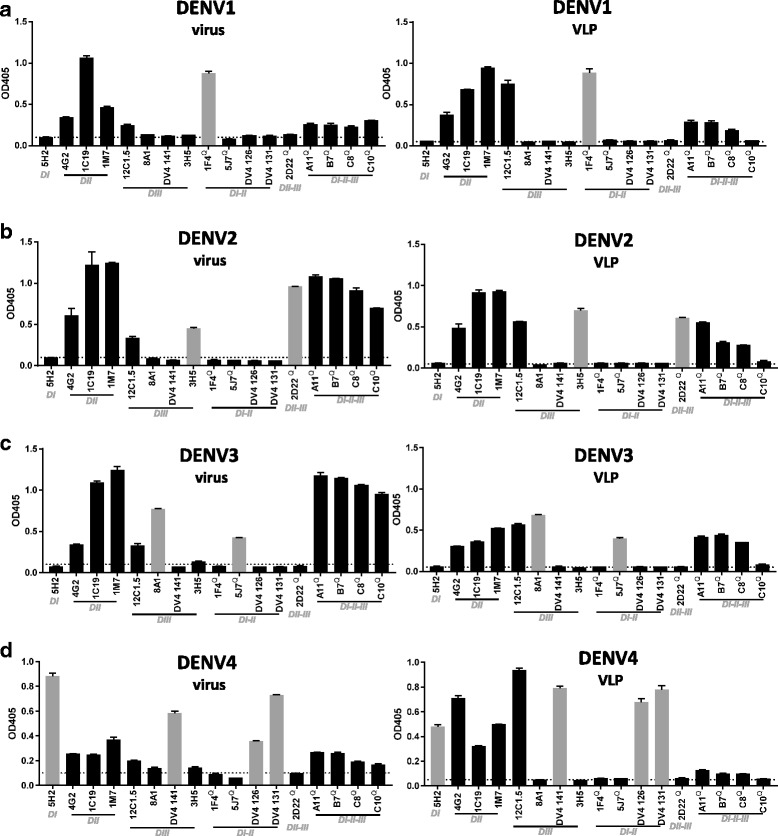
Epitope analysis of DENV1–4 VLPs. **a**-**d** DENV VLPs and virus particles were subjected to a large panel of human and mouse derived, serotype specific (grey bars) or cross-reactive mAbs (black bars) that recognize epitopes of varying complexity (Table 1) by antigen capture ELISA. Quaternary epitope recognizing (Q) mAbs and binding regions are indicated

To compare antibody epitopes displayed by DENV VLPs and infectious virions, we analyzed the binding of a large panel of well-characterized human and mouse derived mAbs (Table 1) to serially diluted VLPs and virions by capture ELISA. Cross-reactive mAbs 4G2 and 1 M7 were used to capture purified virus and VLPs. The tested mAbs vary from serotype specific (TS) or cross-reactive (CR) mAbs that bind epitopes of different complexities and the VLPs and purified fractions were derived from the same batch as used in the CBB, WB and TEM analysis. Figure 3 depicts binding to a virus/VLP concentration of 1.32 ng/μl, which fell in the linear range of mAb binding, which was determined by serial dilutions (data not shown). Binding of the different mAbs to virus antigen can be qualitatively compared to the binding of the mAbs to purified VLPs. For DENV1, binding is observed for the DENV1 TS mAb 1F4 for both virus and VLP samples (Fig. 3a). With the exemption of C10, all CR mAbs such as 1 M7, EDE1 (C8, C10), EDE2 (A11, B7), 1C19, 4G2 and 12C1.5 bind efficiently to DENV1 VLPs. Similar serotype specificity was found for DENV2–4 VLPs. TS mAb 2D22 and 3H5 show specific binding to DENV2 VLPs (Fig. 3b), where binding is absent in the other serotypes. For DENV3, specific binding was observed by 5 J7 and 8A1 (Fig. 3c) and all CR mAbs bound both virus and VLPs. CR binding was observed to a lesser extent for DENV4 VLPs (Fig. 3d). Although the TS mAbs 5H2, DV4 126, DV4 131 and DV4 141 bound DENV4 virus and VLPs accordingly, binding of e.g. EDE mAbs to DENV VLPs was marginal. Interestingly, only minor binding of C10 was observed to any of the VLPs.

Due to the experimental setup, binding signals of the same mAb to virus or VLP cannot be quantifiably compared. However, it is possible to get a relative insight into the differences in epitope exposure and abundance between virus and VLP. For this, we normalized the binding of each mAb to a well-characterized TS, quaternary epitope mAb of each of the 4 serotypes. By comparing the relative binding of each mAb between virus and VLP, one can demonstrate that certain epitopes are better represented on virus particles or VLPs. Future studies are needed for conclusive evidence, it does however give an indication on the relative epitope exposure.

**Fig. 4:**
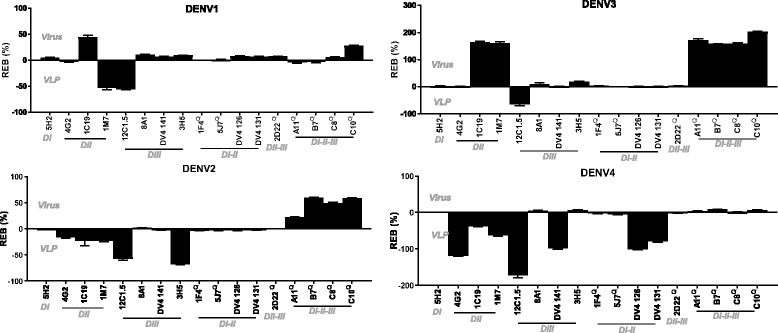
Relative epitope binding on DENV virus vs VLPs. To demonstrate that certain epitopes are better represented on virus particles or VLPs, binding of each mAb within every DENV serotype was normalized to 1F4 (DENV1), 2D22 (DENV2), 5 J7 (DENV3) and 5H2 (DENV4). The normalized binding of each mAb to VLPs was subtracted from the normalized binding to purified virus, resulting in relative epitope binding (REB). Quaternary epitope recognizing (Q) mAbs and binding regions are indicated

Binding of each mAb was normalized to 1F4 (DENV1), 2D22 (DENV2), 5 J7 (DENV3) and 5H2 (DENV4) (Additional file 1). These are well characterized serotype specific mAbs that bind to quaternary epitopes. Normalized binding of each mAb to VLPs was subtracted from the normalized binding to purified virus, resulting in relative epitope binding (REB) (Fig. 4). Relative to 1F4 binding, 1 M7 and 12C1.5 epitopes are better displayed on DENV1 VLPs than on DENV1 virus particles, whereas the C10 and 1C19 epitopes were better displayed on DENV1 virions than on VLPs (Fig. 4a). For DENV2, we observed enhanced binding of the EDE1 and EDE2 mAbs, relative to 2D22. Only 3H5 and 12C1.5 are significantly better recognized on DENV2 VLPs, compared to DENV2 virus (Fig. 4b). 12C1.5 binding was also found to be more proficient on DENV3 VLPs compared to virus particles. 1 M7, EDE mAbs and 1C19 binding was increased on DENV3 virus particles normalized to 5 J7 (Fig. 4c). Interestingly, many of the DV4 TS and CR mAbs such as 4G2 and 12C1.5 bind DENV4 VLPs better than virus particles, when normalized to 5H2.

### DENV infected patient sera is efficiently depleted by DENV VLPs

**Fig. 5:**
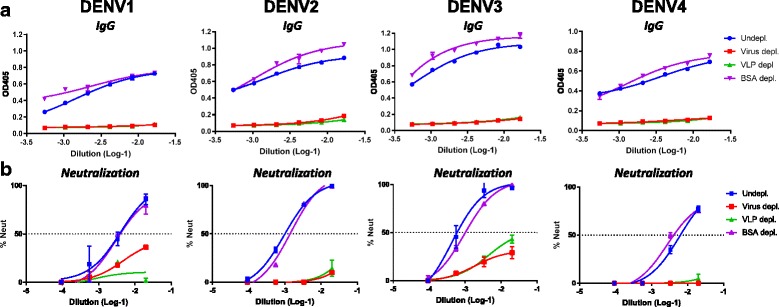
DENV infected patient sera is efficiently depleted by DENV VLPs. Purified DENV particles or DENV VLPs were coated onto magnetic beads and incubated with convalescent patient sera. BSA was used for the depletion handling control. **a** DENV specific IgG levels for all depletion samples were determined by ELISA. **b** DENV specific neutralizing antibody titers were analyzed by a Vero-cell flow cytometry based neutralization assay. Neutralizing titers are expressed as Neut_50_ values indicating the serum dilution where 50% of the virus is neutralized

To characterize infection or vaccine induced immune responses, it is essential to determine if DENV serotype specific or cross-reactive antibodies are responsible for the neutralizing activity in patient sera. These assays are traditionally performed by coating purified infectious virus particles to polystyrene beads, which are subsequently used to capture and deplete different antibody populations from serum samples. Using VLPs instead of infectious virus would offer many practical advantages in these serological assays. We depleted DENV patient convalescent sera (DT000) with DENV VLPs of each serotype individually. DT000 has been infected with multiple DENV serotypes > 20 years ago and has been shown to efficiently neutralize all 4 serotypes [21]. DENV VLPs and purified virus were efficiently crosslinked to magnetic beads and used to deplete DT000 of all DENV specific antibodies. The ability of VLPs to deplete DENV sera was directly compared to that of virus particles (Fig. 5). VLPs of all DENV serotypes depleted DT000 sera equally well as virus particles (Fig. 5a). In accordance with depleted IgG levels, DT000 sera depleted with VLPs has completely lost its neutralizing activity (Fig. 5b).

## Discussion

The immunogenic properties of DENV VLPs have been analyzed in animal models by several groups. However, little is known about E-protein organization on the surface of VLPs [10, 11]. VLP epitope display has not yet been directly compared to that of virus particles and outside the use as vaccine antigens, VLP applicability as serological tools has been underappreciated. The terms subviral particle and VLP are often used to describe the same particle format, but can be considered different. In this study we refer to particles that do not contain a nucleocapsid and are produced after the expression of prM-E as VLPs. In our opinion, the term subviral particle is better used to describe the formation of smaller spherical particles obtained as a side product of wildtype infections.

DENV1–4 VLPs produced in mammalian cells efficiently display epitopes that have mapped on virus particles. TS and CR monoclonal antibodies isolated from different DENV patients with varying infection history bound to the VLPs indicating that native epitopes are present on the VLPs. In the low resolution cryo-EM structural data available on flavivirus virus-like particles or subviral particles, 30 E-dimers are assembled in a *T* = 1 icosahedral lattice, different from the 90 E-dimers found on mature virus particles organized in a *T* = 3 symmetry [12]. Our analysis here demonstrates that overall differences in arrangement of E-dimers on VLPs versus virions and the absence or presence of a nucleocapsid do not alter the display of most quaternary epitopes targeted by human antibodies.

The DENV VLPs were found to have a similar size distribution as the TBEV subviral particles. With particle diameters of ~ 29–34 nm, the VLPs are considerably smaller in size than natural virus particles (~ 50 nm) [4, 12, 22]. The minor VLP size differences between serotypes could be attributed to maturation dissimilarities, however, as the highly concentrated VLPs are not perfect spheres and have structural irregularities, the measurements of diameters might be error prone. Future structural studies are needed to confirm the observed differences in DENV VLP diameter.

Human mAb 1F4 is DENV1 specific and although it only binds the virus and not the E-monomer, its footprint has been mapped within one E-monomer and not across neighboring E-proteins within the homodimer [7]. Despite differences in size and prM content, 1F4 bound equally well to DENV1 VLPs and purified virus. Similar results were found for 2D22 (DENV2), 5 J7 (DENV3) and 5H2 (DENV4). 2D22 is a potent neutralizing antibody and binds epitopes that span across the E-protein dimer, blocking envelope reorganization necessary for viral fusion [5]. DENV3 specific mAb 5 J7 has a footprint that spans across neighboring E-dimers including residues at the EDI/EDII hinge region [23]. Binding of 5 J7 to DENV3 VLPs strongly indicates that E-dimers interact and form poly-dimer structures similar to the rafts found on virion surfaces. For long, 5H2 was the only DENV4 specific highly neutralizing mAb of which the binding footprint has been mapped. This chimpanzee derived mAb docks within EDI of E [24]. It does not bind monomeric E-proteins, but efficiently binds both DENV4 virus and VLPs. Recent studies have identified new DENV4 TS mAbs that bind E-protein in ranging complexity [8]. DV4 126 and DV4 131 are highly neutralizing and their quaternary footprint was mapped to the EDI/EDII hinge region. Just like 5 J7, these mAbs do not bind monomeric E-proteins, but recognize virus and VLPs.

We used 1F4, 2D22, 5 J7 and 5H2 to normalize the binding of all the other mAbs to both virus and VLP, allowing for a relative comparison between epitope availability on virus vs VLPs. Relative to these mAbs we see that the EDIII binding mAbs 12C1.5 and 3H5 bind better to VLPs, indicating that the EDIII might be more exposed on VLPs. The quaternary CR EDE epitopes are relatively better presented on DENV2–3 virus particles. In particular EDE1-C10 binding has a strong preference for virus particles, due to the absence of binding to VLPs of all serotypes. It remains unclear why C10 does not bind VLPs, while the other EDE mAbs, that share a very similar footprint, bind efficiently.

The maturity of virus particles and VLPs affects epitope display as exemplified by the fusion loop binding CR mAb 4G2. During virus replication, the pr peptide associates with the fusion loop in E, shielding it from premature low-pH induced fusion with host cell membranes [25]. Our analysis indicates that the fusion loop is efficiently exposed on DENV4 VLPs, which is in accordance to the undetectable prM levels in DENV4 VLPs. Differences in prM levels between DENV2–3 VLPs and virus might have similar steric hindrance effects on the EDE mAbs. However, this requires further structural analysis. In general, epitopes present on the fusion loop domain, EDIII and DI/DII/DIII spanning regions are all represented on DENV VLP surfaces.

The footprints of many mAbs have been mapped on dimers generated from soluble monomeric E proteins. Soluble E monomers crystallize into dimers and do not contain prM. Even though it is assumed that the crystallized dimers are structurally similar to E-dimers found on virus particles, they might differ when part of complex multiprotein complexes on VLP envelopes. Future studies should focus on DENV VLP protein structures to answer these questions.

The VLPs and virus particles were captured by mAbs 4G2 and 1 M7, suggesting a bias for fusion loop exposed particles. However, using other capturing mAbs, the bias would be skewed towards particles displaying that specific epitope. Using other well-characterized mAbs, we have shown that DENV VLPs display an epitope landscape very similar to that found on virus particles. Depletion studies using VLPs as a depletion antigen translate this finding to polyclonal sera of DENV infected patients. Secondary infected serum was efficiently depleted from all TS and CR (non)-neutralizing antibodies. Serum depletions are usually performed using infectious purified virus antigen conjugated to microbeads. However the use of VLPs has practical benefits over the use of purified antigen. Issues of infectious virus production and purification and virus leaching into the serum during handling are not present when using VLPs. Additionally, VLPs are more easily genetically adapted and are therefore valuable tools in flavivirus pAb and mAb mapping and serology.

## Conclusions

Flavivirus VLP based vaccines are amongst the leading candidates in ongoing vaccine studies. Understanding the structure of VLPs is essential for interpreting the antibody response in vaccinees. VLPs also have potential value as diagnostic reagents. Besides, VLPs can be produced as a byproduct of natural infection. Therefore, the structure of VLPs also becomes important from the standpoint of virus assembly. By exploiting antibodies and insights high resolution structure of dengue virions, this study aids in further understanding the antigenic landscape of DENV VLPs and presents a comparative antigenic surface view of VLPs in respect to virus particles.

## Additional file

Additional file 1: Normalized antibody binding to DENV virus particles and VLPs. The binding of each mAb to virus (black bars) or VLPs (grey bars) within every DENV serotype was normalized to 1F4 (DENV1), 2D22 (DENV2), 5 J7 (DENV3) and 5H2 (DENV4). (PDF 220 kb)
