# Supplementary material for: Dengue virus-like particles mimic the antigenic properties of the infectious dengue virus envelope
Source: Virol J. 2018 Apr 2;15:60. doi: 10.1186/s12985-018-0970-2 (PMC5879749; doi:10.1186/s12985-018-0970-2)
Supplement: Supplementary file 1 — Normalized antibody binding to DENV virus particles and VLPs. The binding of each mAb to virus (black bars) or VLPs (grey bars) within every DENV serotype was normalized to 1F4 (DENV1), 2D22 (DENV2), 5 J7 (DENV3) and 5H2 (DENV4). (PDF 220 kb) [file 12985_2018_970_MOESM1_ESM.pdf]

Additional file 1.

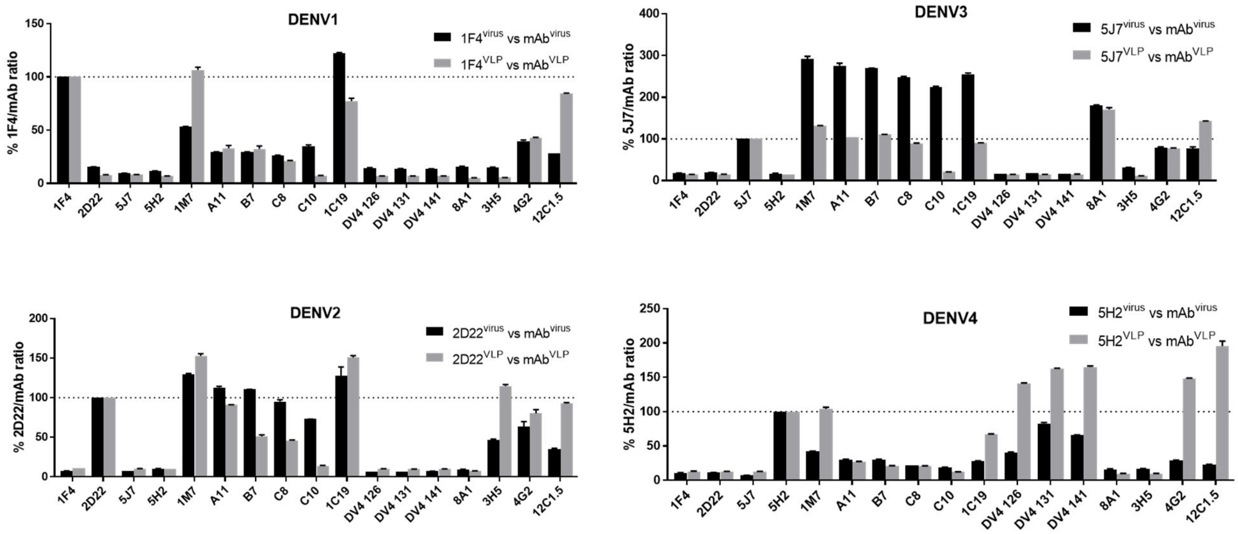

Additional file 1. Normalized antibody binding to DENV virus particles and VLPs. The binding of each mAb to virus (black bars) or VLPs (grey bars) within every DENV serotype was normalized to 1F4 (DENV1), 2D22 (DENV2), 5J7 (DENV3) and 5H2 (DENV4).
